# 

**Published:** 1860-04

**Authors:** 


Vol. I.] APRIL, 1860. [No. 4.

### THE CHICAGO

### MEDICAL EXAMINER

A Monthly Journal, devoted to the Educational, Scientific, and practical interests of the Medical Profession.

EDITED BY

N. S. DAVIS, M. D.

Professor of Principles and Practice of Medicine and Clinical Medicine, in the Medical Department of Lind University,

AND

E. A. STEELE, M. D.

Terms—$2.00 per annum, in advance.

CHICAGO:

Wm. Cravens & Co. Printers and Publishers,

No. 132 LAKE STREET.

